# Doubly bi-allelic variants of *MTHFR* and *MTHFD1* in a Chinese patient with hyperhomocysteinemia and failure of folic acid therapy

**DOI:** 10.3389/fgene.2022.964990

**Published:** 2023-01-04

**Authors:** Yu-Xing Liu, Man-Hua Ding, Yue Sheng, Meng-Fei Sun, Lv Liu, Yang Zhang

**Affiliations:** ^1^ Department of Neurology, Xuzhou Central Hospital, Xuzhou, China; ^2^ Department of Cell Biology, School of Life Sciences, Central South University, Changsha, China; ^3^ Department of Radiotherapy, Xuzhou Cancer Hospital, Xuzhou, China; ^4^ Department of Respiratory Medicine, Diagnosis and Treatment Center of Respiratory Disease, The Second Xiangya Hospital of Central South University, Changsha, China

**Keywords:** *MTHFR*, *MTHFD1*, hyperhomocysteinemia, folic acid, whole-exome sequencing

## Abstract

**Background:** Hyperhomocysteinemia (HHcy) is a risk factor for thromboembolic disease. Defects in one-carbon metabolism (1-CM)-related genes, such as methylenetetrahydrofolate reductase (*MTHFR*), methylenetetrahydrofolate dehydrogenase, cyclohydrolase, and formyltetrahydrofolate synthetase 1 (*MTHFD1*), can cause HHcy and may also affect the efficacy of folic acid therapy. The details of mechanisms are yet to be further investigated.

**Method:** We described a Chinese family with hereditary HHcy. The proband suffered from severe thromboembolic disease and experienced failure of folic acid therapy. Two sons of the proband were also diagnosed with HHcy but were sensitive to folic acid therapy. Whole-exome sequencing (WES) was conducted to evaluate the genetic lesion of this family.

**Results:** Compound heterozygous variants (a common polymorphism, p. A222V, and a novel variant, p. C631*fs*1) of the *MTHFR* gene and a homozygous missense variant (p. K134R) of the *MTHFD1* gene were identified in the proband. The two sons, with successful intervention, only harbored the homozygous p. A222V variant of the *MTHFR* gene.

**Conclusion:** The clinical manifestations and genetic research synergistically confirmed the diagnosis of HHcy and clarified the failure of folic acid therapy in the proband caused by doubly bi-allelic variants of the *MTHFR* and *MTHFD1* genes. Our study increased our understanding of the molecular basis of 1-CM-related gene defects on folic acid therapy in HHcy.

## Introduction

Hyperhomocysteinemia (HHcy) refers to abnormally high levels of plasma/serum homocysteine (Hcy) and is a risk factor for thromboembolic disease with highly variable symptoms including atherosclerosis, cerebral infarction, and pulmonary embolism (Son and Lewis, 2022). Some brain-related disorders, including epilepsy, have also been reported to be associated with HHcy (Hrncic et al., 2018). At present, folate supplementation is a common treatment for HHcy, while the efficacy may be affected by many factors (Du et al., 2018; Li et al., 2020). The invalid response to folate supplementation treatment remains a clinical challenge (Li et al., 2020).

The plasma Hcy level is closely associated with one-carbon metabolism (1-CM) which comprises methionine and Hcy metabolism. Genetic deficiencies in 1-CM can cause inherited HHcy, such as the methionine synthase deficiency, folic acid synthesis deficiency, methylenetetrahydrofolate reductase (MTHFR) deficiency, and methylenetetrahydrofolate dehydrogenase, cyclohydrolase, and formyltetrahydrofolate synthetase 1 (MTHFD1) deficiency (Burda et al., 2015a; Lyon et al., 2020; Steele et al., 2020; Biesalski et al., 2022; Sudheer et al., 2022). MTHFR deficiency results in homocystinuria and HHcy in an autosomal recessive pattern, and p. A222V of the *MTHFR* gene in the homozygous state is the most common genetic cause of elevated plasma Hcy. Compound heterozygous variants combined by the p. A222V mutation and other mono allelic *MTHFR* variants have also been reported in HHcy cases (Rummel et al., 2007; Clement et al., 2022; Fatima et al., 2022). Similarly, patients with MTHFD1 deficiency may present with HHcy merged with other phenotypes, including megaloblastic anemia and hemolytic uremic syndrome (Burda et al., 2015a). Compared with the *MTHFR* variant, few *MTHFD1* mutations are identified in HHcy cases. Genetic polymorphisms of key enzymes in 1-CM, including betaine–homocysteine methyltransferase (*BHMT*), *MTHFR*, *MTHFD1*, methionine synthase reductase (*MTRR*), and methionine synthase (*MTR*), have also been reported to affect the efficacy of folate therapy in HHcy (Du et al., 2018; Li et al., 2019; Partearroyo et al., 2019). The details of mechanisms are yet to be further investigated.

Here, we reported a Chinese family with hereditary HHcy. The proband suffered from severe thromboembolic disease, and folic acid therapy was ineffective. Two sons of the proband were also diagnosed with HHcy but benefitted from folic acid therapy. Whole-exome sequencing (WES) was conducted to evaluate the genetic lesion of this family and identified doubly bi-allelic variants in *MTHFR* and *MTHFD1* genes. Among these variants, the variant (p.C631*fs*1) of the *MTHFR* gene has not been published previously, and therefore, it is considered to be a novel mutation.

## Materials and methods

### Ethical approval

This study was approved by the Review Board of Xiangya Hospital, Changsha, China. Written informed consent was obtained from all adult participants and legal guardians of minor participants.

### Subjects

All family members (three patients and one healthy member) were enrolled and diagnosed by ultrasound examination, coronary angiogram, and laboratory inspection. The clinical data, including folate acid, vitamin B12 (VB12), ferritin, and erythropoietin (EPO), of each family member were collected for diagnosis or response evaluation. A total of 200 healthy subjects, as described in our previous study, were enrolled in this study to exclude polymorphisms (Wang et al., 2021).

### Whole-exome sequencing

Genomic DNA was prepared from peripheral blood lymphocytes of all the participants using the DNeasy Blood & Tissue Kit (QIAGEN, Valencia, CA, United States). The primary sequence for WES was provided by the Novogene Bioinformatics Institute (Beijing, China). Exomes were captured using the SureSelect Human All Exon V6 kit (Agilent, Santa Clara, CA, United States), and next-generation sequencing was conducted using a HiSeq X Ten system (Illumina, San Diego, CA, United States). The strategies of data filtering and the necessary bioinformatics analyses can be referred to in the previous studies published by our laboratory group (Fan et al., 2019; Wang et al., 2021).

### Mutation validation and co-segregation analysis

Sanger sequencing was used to validate the candidate variants identified in WES. Segregation analysis was performed in the family members of this study. Sequences of the polymerase chain reaction (PCR) products were determined using the ABI 3100 Genetic Analyzer (ABI, Foster City, CA, United States).

### Bioinformatics analysis

SWISS-MODEL software (https://swissmodel.expasy.org/interactive) was used to identify the function of the mutation. The conservation analysis was performed by ConSurf Server software (http://consurf.tau.ac.il/).

## Results

### Clinical description

**FIGURE 1:**
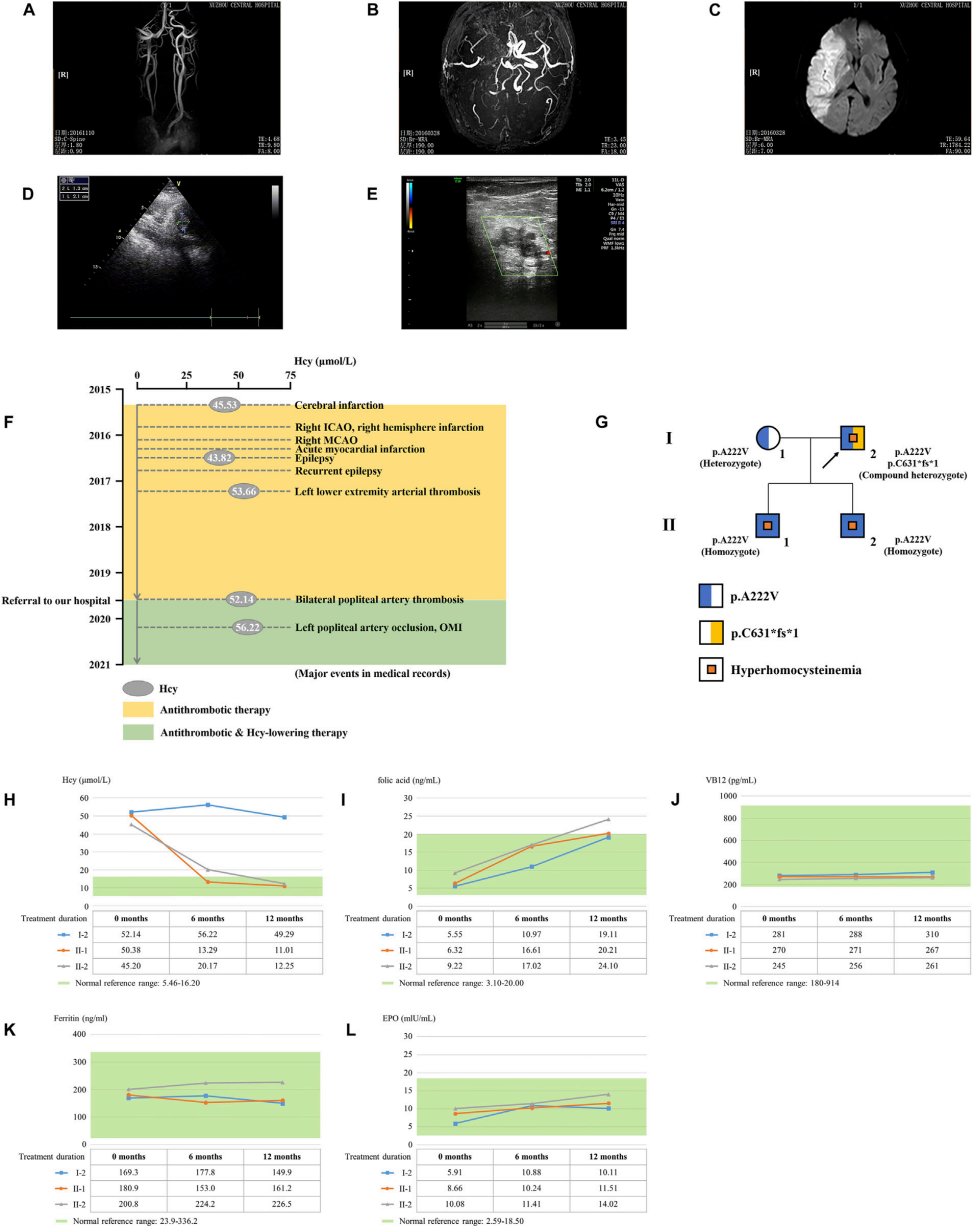
Clinical data on this HHcy family. The head MRA showed the **(A)** right internal carotid artery, **(B)** middle cerebral artery, and **(C)** right cerebral hemisphere of the proband (I-2). **(D)** Cardiac color ultrasound showed the left ventricular mural thrombosis of the proband. **(E)** Ultrasound examination showed lower-limb artery thrombosis of the proband. **(F)** Major events in the medical records of the proband. ICAO, internal carotid artery occlusion; MCAO, middle cerebral artery occlusion; OMI, old myocardial infarction. **(G)** Genealogy of this HHcy family. Squares indicate male family members; circles, female members; and arrow, proband. **(H)** Time course of Hcy-lowering therapy. The changes of Hcy, **(I)** folic acid, **(J)** VB12, **(K)** ferritin, and **(L)** EPO during treatment.

The proband is a 51-year-old farmer from Jiangsu Province in China. He was referred to our hospital due to the progression of severe thrombotic disease. A medical history investigation showed that he suffered from cerebral infarction combined with secondary epilepsy, old myocardial infarction (OMI), and lower extremity arterial thrombosis. He was a non-smoker and had no previous history of diabetes, hypertension, or traumatic head injury. The head magnetic resonance angiography (MRA) showed severe stenosis of the right internal carotid artery and middle cerebral artery, and large softening lesions appeared in the right cerebral hemisphere (Figures 1A–C). A left ventricular mural thrombus was found in the proband (Figure 1D), as well as thrombosis in the left lower extremity artery and bilateral popliteal artery (Figure 1E). Laboratory examination showed a much higher level of total plasma Hcy (52.14 μmol/L), while the results for folate acid (5.55 ng/ml), VB12 (281 pg/ml), ferritin (169.3 ng/ml), and EPO (5.91 mIU/ml) were normal. Financial reasons prevented the patient from further hospitalization for testing and treatment after his condition improved slightly in the previous treatment. The major events in the medical records during 2016–2019 are shown in Figure 1F with several available data of Hcy. The proband was diagnosed with HHcy, and his thrombotic disease was related to a high level of plasma Hcy. A family history survey showed that two sons of the proband, II-1 and II-2, also had high levels of plasma Hcy. The eldest son (II-1) had developed pulmonary embolism and deep venous thrombosis of the right lower limb since he was 30 years old. The proband’s wife (I-1) had a normal plasma–Hcy level (Figure 1G). The levels of folate and VB12 detected in three family members (I-1, II-2, and II-3) were normal, denying the relationship between the abnormal Hcy level and a lack of folic acid or VB12 in this family and putting forward the possibility of genetic factors. No other malformations were detected in this family (Table 1). Under the diagnosis of inherited HHcy, all patients in this family were managed with folate (5 mg/day), thiamine (75 mg/day), and mecobalamin (1.5 mg/day), which decreased the two sons’ plasma–Hcy level to normal for 1 year after the diagnosis. Confusingly, the proband’s plasma–Hcy level remained elevated (49.29–52.14 μmol/L) despite 1 year of medications. Changes of various indexes, including Hcy, folic acid, VB12, ferritin, and EPO, during treatment are shown in Figures 1H–L.

**TABLE 1:** Clinical data on the members in this family.

Subject	I-1	I-2 (proband)	II-1	II-2	Note
Sex	F	M	M	M	-
Age (years)	49	51	32	29	-
Hcy (μmol/L)	11.14	52.14	50.38	45.20	NRR: 5.46-12.60
Folic acid (ng/ml)	6.98	5.55	6.32	9.22	NRR: 3.10-20.00
VB12 (pg/ml)	302	281	270	245	NRR: 180-914
Ferritin (ng/ml)	177.9	169.3	180.9	200.8	NRR: 23.9-336.2
EPO (mlU/mL)	6.15	5.91	8.66	10.08	NRR: 2.59-18.50
Cerebral infarction	−	+	−	−	−
Epilepsy	−	+	−	−	−
Myocardial infarction	−	+	−	−	−
Lower extremity arterial thrombosis	−	+	+	NA	−
Pulmonary embolism	−	−	+	−	−
HHcy	−	+	+	+	−
Failure of folic acid therapy	−	+	−	−	−

Abbreviations: F, female; Hcy, homocysteine; HHcy, hyperhomocysteinemia; M, male; NA, not available; VB12, vitamin B12; EPO, erythropoietin.

### Genetic analysis

**FIGURE 2:**
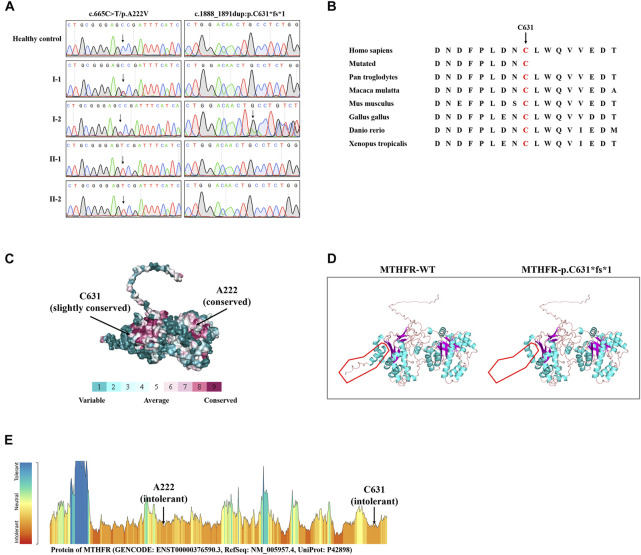
Sequencing data and bioinformatics analysis of *MTHFR* variants. **(A)** Sanger sequencing results of the *MTHFR* variants in this family. **(B)** Alignment of multiple *MTHFR* protein sequences across species. Letters in red show the C631 sites that are evolutionarily conserved. **(C)** Conservation analysis of the D612 amino acid was predicted by ConSurf Server software. **(D)** Structural prediction of the mutant protein. The wild-type *MTHFR* (*MTHFR*-WT) protein structure and the p. C631*fs*1 mutant *MTHFR* (*MTHFR*-p.C631*fs*1) protein structure were predicted by SWISS-MODEL online software. **(E)** MetaDome server was used to identify the intolerant regions in *MTHFR*. As depicted, the affected nucleotides/residues are located in the intolerant region.

WES yielded 10.82 GB of data with 97.14% coverage of the target region, and 95.75% of the target covered over 10×. Totally, about 79,924 variants were detected in the proband. After data filtering, a previously not described heterozygous mutation (c.1888_1891dup; p.C631*fs*1) was identified in exon 12 of the *MTHFR* gene. In addition, another known heterozygous variant (c.665C>T; p. A222V, known as c. 677C>T; p. A222V) was also identified in exon 5 of the *MTHFR* gene (Fatima et al., 2022). Sanger sequencing was performed on all the family members. Co-segregation analysis showed that two sons of the proband (II-1 and II-2) harbored the homozygous p. A222V variant of the *MTHFR* gene. The proband’s wife (I-1) was healthy and harbored the heterozygous p. A222V variant of the *MTHFR* gene (Figures 1G, 2A). Thus, the patients of HHcy in this family were further diagnosed with congenital *MTHFR* deficiency. Alignment of *MTHFR* amino acid sequences revealed that the affected amino acid C631 is conserved in different species (Figure 2B). At the same time, ConSurf Server software (http://consurf.tau.ac.il/) predicted that two affected amino acids were evolutionarily conserved (Figure 2C). In addition, SWISS-MODEL software (https://swissmodel.expasy.org/) showed that the mutation at C631 may lead to a loss of C-terminal in the mutated *MTHFR* protein (Figure 2D). Furthermore, Metadome software (https://stuart.radboudumc.nl/metadome/dashboard) indicated that two affected residues are located in the intolerant region of *MTHFR* (Figure 2E).

**FIGURE 3:**
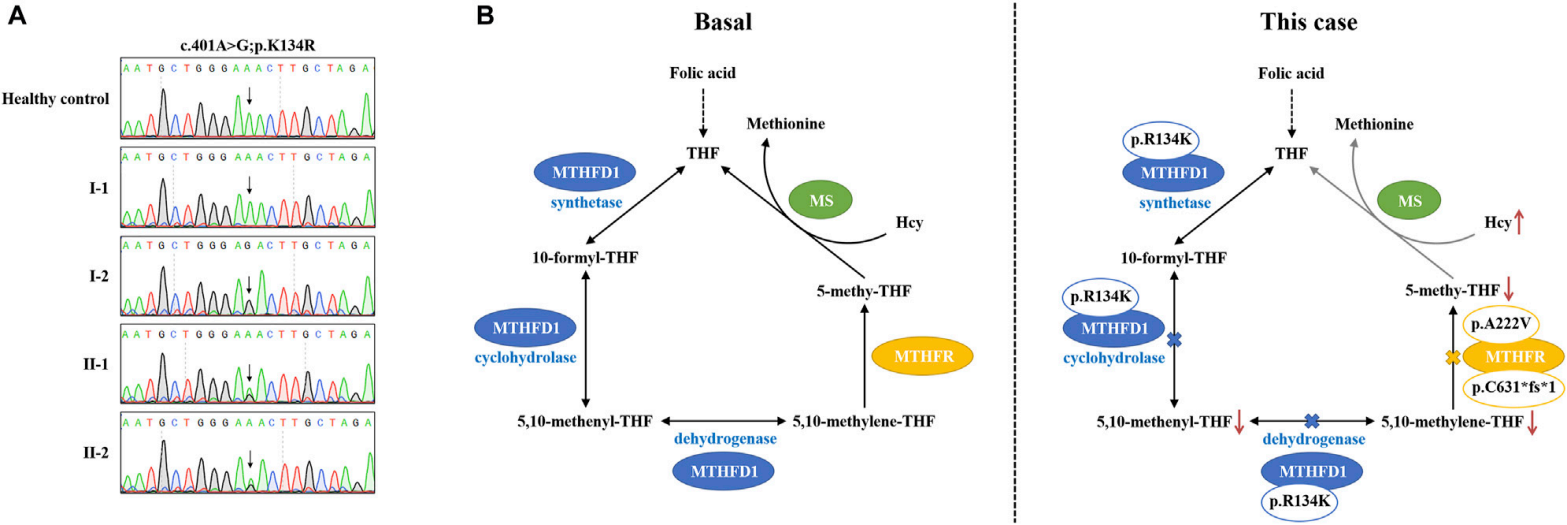
Pathogenesis hypothesis of the proband. **(A)** Sequencing data on *MTHFD1* variants in this family. **(B)** Both MTHFD1 and MTHFR participated in the cytoplasmic folate cycle. Three reactions are catalyzed by *MTHFD1*: the conversion of formate and THF to 10-formyl-THF (synthetase); the interconversion of 10-formyl-THF and 5,10-methenyl-THF (cyclohydrolase); and the reduction of 5,10-methenyl-THF to 5,10-methylene-THF (dehydrogenase). *MTHFR* catalyzes the reduction of 5,10-methylene-THF to 5,10-methenyl-THF. 5,10-Methenyl-THF is utilized by methionine synthase to convert homocysteine to methionine. In our case, the p. R134K variant is located within the dehydrogenase/cyclohydrolase domain of the *MTHFD1* protein which may lead to reduction of 5,10-methenyl-THF and 5,10-methylene-THF syntheses. On the other hand, the compound heterozygous variants, p. A222V and p. C631*fs*1, may result in dysfunction of the *MTHFR* enzyme, causing reduction of the 5,10-methenyl-THF synthesis. As a result, the conversion of homocysteine to methionine is inhibited by the low level of 5,10-methenyl-THF, which may contribute to the abnormal accumulation of plasma–Hcy and finally lead to HHcy and failure of folic acid therapy, as observed in our patient.

The plasma–Hcy levels of two sons were successfully intervened after folic acid therapy, while the proband’s plasma–Hcy level remained elevated despite 1 year of medications. Since genetic polymorphisms of key enzymes in 1-CM may affect the efficacy of folate therapy in patients with HHcy (Du et al., 2018), we further investigated other possibilities underlying his Hcy level besides compound heterozygous *MTHFR* variants. The results indicated that the patient carried an additional homozygous variant (c.401A>G; p.K134R) of the *MTHFD1* gene. Sanger sequencing confirmed that two sons of the proband (II-1 and II-2) harbored this heterozygous variant; this variant was not detected in the proband’s wife (I-1) (Figure 3A).

## Discussion

In this study, we reported a Chinese family with hereditary HHcy. The proband suffered from severe thromboembolic disease and failed to accept folic acid therapy. Two sons of the proband were diagnosed with HHcy but were sensitive to folic acid therapy. Exome sequencing was conducted to evaluate the genetic cause in this family. Simultaneously, compound heterozygous variants and a homozygous variant of the *MTHFR* gene were identified in the HHcy individuals (I-2, II-1, and II-2) of this family. Furthermore, an additional homozygous variant of the *MTHFD1* gene was identified in the proband (I-2) within whom folic acid therapy was invalid. Our study suggests that doubly bi-allelic variants of the *MTHFR* and *MTHFD1* genes are associated with HHcy and caused a failure of folic acid therapy.

MTHFR is considered as the key enzyme in controlling the plasma–Hcy levels (Rummel et al., 2007). It catalyzes an unusual two-step reaction. The first step is reducing the equivalents that are transferred from nicotinamide adenine dinucleotide phosphate (NADPH) to the cofactor flavin adenine dinucleotide (FAD). The second step is reducing equivalents that were further passed on to 5,10-methylenetetrahydrofolate (5,10-methylene-THF), forming 5-methyltetrahydrofolate (5-methyl-THF) (Guenther et al., 1999). 5-Methyl-THF is the predominant circulatory form of folate and is also the major carbon donor in the remethylation of homocysteine to methionine. Thus, the MTHFR enzyme is essential for maintaining the pool of circulating folate and methionine and reducing the levels of homocysteine (Zaric et al., 2019; Raghubeer and Matsha, 2021). The MTHFR protein is composed of an N-terminal catalytic domain and a C-terminal regulatory domain. The binding sites for the substrates NADPH, 5,10-methylene-THF, and the co-factor FAD are located in the N-terminal catalytic domain (Guenther et al., 1999). A previous study has indicated that missense mutations in the N-terminal catalytic domain may lead to a loss of *MTHFR* activity, whereas in the C-terminal regulatory domain, only truncating mutations have such a severe effect (Burda et al., 2015b). The novel frameshift variant p. C631*fs*1 identified in this study is located in the C-terminal regulatory domain, leading to a truncated MTHFR protein. Although the change of the *MTHFR* enzyme activity has not been assayed, we presumed this variant is pathogenic, according to previous studies. Homozygosity for the p. A222V variant is the most common genetic cause of HHcy (Guenther et al., 1999), and A222V substitution is a known cause for reduced levels of specific activity and increased thermolability. Furthermore, compound heterozygous p. A222V variant and another *MTHFR* gene variant have also been reported in intermediate HHcy subjects (Rummel et al., 2007). Consistent with previous reports, we reported an HHcy patient with the compound heterozygous state for the known variant p. A222V and a novel variant of p. C631*fs*1 in the *MTHFR* gene. A previous study also indicated that the p. A222V variant combined with severe *MTHFR* mutations in the compound heterozygous state may require further therapy in addition to folate substitution to normalize homocysteine levels (Rummel et al., 2007), which is similar to the features in this proband. Notably, the mutation (p. C631*fs*1) detected in this research has not been published, and therefore, it is considered novel.

MTHFD1 is a tri-functional protein comprising 5,10-methenyltetrahydrofolate (5,10-methenyl-THF), cyclohydrolase, 5,10-methylene-THF dehydrogenase, and 10-formyltetrahydrofolate (10-formyl-THF) synthetase. As a 1-CM-related enzyme, MTHFD1 catalyzes three reactions involved in folate metabolism and plays an important role in Hcy metabolism (Christensen et al., 2009; Khaki et al., 2019; Bidla et al., 2020). MTHFD1 is encoded by the gene *MTHFD1*. Homozygous mutations at *MTHFD1* may lead to disorders of the folate-mediated Hcy pathway and could affect folate treatment response (Bidla et al., 2020; Li et al., 2020). The p. R134K variant (rs1950902) is a common non-synonymous SNP in the *MTHFD1* gene. Epidemiological studies have reported a connection between the p. R134K variant, and diseases relate to folate metabolism or Hcy levels (Sutherland et al., 2014). A recent study illustrated that rs1950902 significantly affected changes in the Hcy level and further indicated the relevance of rs1950902 in the folate treatment response (Li et al., 2020), although in the absence of functional consequences (Sutherland et al., 2014). In our study, the HHcy proband failed to reach the normal Hcy range after 1 year of folate supplementation. Aside from compound heterozygous variants in the *MTHFR* gene, an additional homozygous variant (p.K134R) of the *MTHFD1* gene was detected. The p. R134K variant, which is located within the dehydrogenase/cyclohydrolase domain of the *MTHFD1* protein, may affect the biosynthesis of 5,10-methenyl-THF and 5,10-methylene-THF (Burda et al., 2015a). In this scenario, we hypothesize the pathogenesis that doubly bi-allelic variants of *MTHFR* and *MTHFD1* lead to a reduction of 5,10-methenyl-THF/5,10-methylene-THF synthesis when combined with dysfunctional MTHFR, which eventually results in HHcy and the failure of folic acid therapy presented in our patient (Figure 3B). Although this suspicion will only be proven by further study, additional functional analysis of the doubly bi-allelic variants is recommended and may result in additional information about the pathogenetic mechanism of HHcy. Based on the genetic findings, the proband was further managed with betaine (6 g/day), thiamine (75 mg/day), mecobalamin (1.5 mg/day), and 5-methyl-THF (600 mg/day) (Guenther et al., 1999; Servy et al., 2018). A follow-up visit has also been scheduled to ensure the patients’ benefit from personalized treatment.

## Conclusion

We used WES to evaluate the genetic cause in a Chinese family with hereditary HHcy and revealed that doubly bi-allelic variants of *MTHFR* and *MTHFD1* genes can result in an HHcy phenotype with failure of folic acid therapy. Our study provided data for genetic counseling and personalized treatment to this family, which might contribute to a more comprehensive genetic counseling in HHcy patients, and further demonstrated the important role for 1-CM in HHcy control.

## Data Availability

The data presented in the study are deposited in the GSEHuman repository, accession number HRA003374 (https://ngdc.cncb.ac.cn/gsa-human/browse/HRA003374).
